# Summertime Tintinnid Community in the Surface Waters Across the North Pacific Transition Zone

**DOI:** 10.3389/fmicb.2021.697801

**Published:** 2021-08-11

**Authors:** Haibo Li, Jun Xuan, Chaofeng Wang, Zhaohui Chen, Gérald Grégori, Yuan Zhao, Wuchang Zhang

**Affiliations:** ^1^CAS Key Laboratory of Marine Ecology and Environmental Sciences, Institute of Oceanology, Chinese Academy of Sciences, Qingdao, China; ^2^Laboratory for Marine Ecology and Environmental Science, Qingdao National Laboratory for Marine Science and Technology, Qingdao, China; ^3^Center for Ocean Mega-Science, Chinese Academy of Sciences, Qingdao, China; ^4^University of Chinese Academy of Sciences, Beijing, China; ^5^Physical Oceanography Laboratory/Frontiers Science Center for Deep Ocean Multispheres and Earth System, Ocean University of China, Qingdao, China; ^6^Aix-Marseille University, Université de Toulon, CNRS, IRD, Mediterranean Institute of Oceanography, Marseille, France

**Keywords:** tintinnids, transition zone species, community, variation, North Pacific Transition Zone

## Abstract

Located from 35° to 45° latitude in both hemispheres, the transition zone is an important region with respect to the planktonic biogeography of the sea. However, to the best of our knowledge, there have been no reports on the existence of a tintinnid community in the transition zone. In this research, tintinnids along two transects across the North Pacific Transition Zone (NPTZ) were investigated in summer 2016 and 2019. Eighty-three oceanic tintinnid species were identified, 41 of which were defined as common oceanic species. The common oceanic species were further divided into five groups: boreal, warm water type I, warm water type II, transition zone, and cosmopolitan species. *Undella californiensis* and *Undella clevei* were transition zone species. Other species, such as *Amphorides minor*, *Dadayiella ganymedes*, *Dictyocysta mitra*, *Eutintinnus pacificus*, *Eutintinnus tubulosus*, *Protorhabdonella simplex*, and *Steenstrupiella steenstrupii*, were the most abundant in the NPTZ but spread over a much larger distribution region. Species richness showed no obvious increase in the NPTZ. Boreal, transition zone, and warm water communities were divided along the two transects. Tintinnid transition zone community mainly distributed in regions with water temperatures between 15 and 20°C. The tintinnid lorica oral diameter size classes were dominated by the 24–28 μm size class in three communities, but the dominance decreased from 66.26% in the boreal community to 48.85% in the transition zone community and then to 22.72% in the warm water community. Our research confirmed the existence of tintinnid transition zone species and community. The abrupt disappearance of warm water type I species below 15°C suggested that this group could be used as an indicator of the northern boundary of the NPTZ.

## Introduction

The study on planktonic biogeography showed that generally, there are nine approximately parallel belts of plankton groups depending on latitude (Longhurst, 2007). Two of these belts are located between 35° and 45° latitude in both hemispheres and are transition zones (Pearcy, 1991; Longhurst, 2007). There have been reports on some plankton species that restricted in transition zones, such as phytoplankton (Venrick, 1971; Semina, 1997), mesozooplankton, including euphausiids (Brinton, 1962; Johnson and Brinton, 1963), chaetognaths (Johnson and Brinton, 1963), copepods and pteropods (Brinton, 1962; McGowan, 1971), and protozoa, including foraminifera (Boltovskoy and Correa, 2016b) and radiolaria (Boltovskoy and Correa, 2016a; Zhang et al., 2018). There are also some plankton species with a wider area of distribution but with the greatest abundance in transition zones.

The North Pacific Transition Zone (NPTZ) lies between the subarctic and subtropical gyres at approximately 30–32°N and 42–45°N in the central Pacific (Roden, 1991; Chai et al., 2003). The warm Kuroshio Current and the cold Oyashio Current converge and generate sharp changes in thermohaline structure, hydrostatic stability structure, and biological species composition in this zone (Roden, 1991; Polovina et al., 2001; Church et al., 2008; Follett et al., 2021). The latitudinal position and physical characteristics of the NPTZ progress seasonally (Church et al., 2008; Howell et al., 2012; Follett et al., 2021; Navarra and Di Lorenzo, 2021) and also present interannual variations (Polovina et al., 2017; Yamaguchi et al., 2017). Normally, the northern boundary of NPTZ is the Subarctic Front with typical characteristic of 33 isohaline (Chai et al., 2003), while the southern boundary is the Subtropical Front, which is defined as the surface outcropping of the 17°C isotherm and 34.8 isohaline (Roden, 1991; Chai et al., 2003). The NPTZ delineate species distribution and is considered as a boundary of planktonic species. Phytoplankton and zooplankton biomass shows an extremely steep latitudinal gradient in this zone (McGowan and Williams, 1973). Previous studies also showed that some planktons occurred only north or south of the NPTZ, while some species were found almost exclusively in the NPTZ (Roden, 1991; Yamaguchi et al., 2017).

Tintinnids are single-cell protozoan planktons that live in marine and freshwater environments. Taxonomically, tintinnids belong to the subclass Choreotrichia, class Spirotrichea, and phylum Ciliophora (Lynn, 2008). They are grazers of pico- and nanoplankton and prey of mesozooplankton (Stoecker and Capuzzo, 1990; Dolan, 2010). Therefore, tintinnids are an important linkage between the microbial food web and traditional food chain. Biogeographically, tintinnids were divided into neritic, warm water, boreal, austral, and cosmopolitan types based on their distribution in the global ocean (Dolan et al., 2013b). Neritic tintinnids mainly living in nearshore and coastal waters and seldom appeared in open seas. Warm water, boreal, austral, and cosmopolitan tintinnids that mainly distribute in open seas were considered as oceanic species (Dolan et al., 2013b). Thus, distinct tintinnid communities should exist from subarctic across the NPTZ to subtropical regions in the North Pacific. However, to the best of our knowledge, data of tintinnid community variations in the NPTZ are scarce. In this study, we investigated the surface water tintinnid community across the western part of the NPTZ to determine (1) whether there are tintinnid transition species within the NPTZ and (2) the characteristics of tintinnid transition zone community.

## Materials and Methods

### Study Area and Sample Collection

**FIGURE 1:**
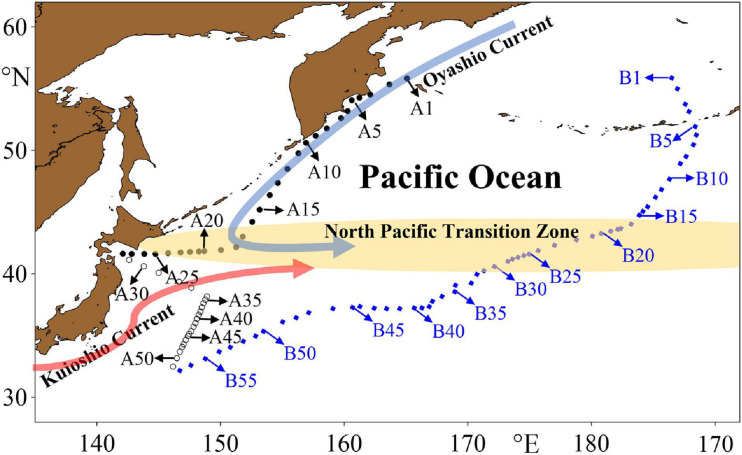
Locations of sampling stations and a schematic of summer circulation in the research area. 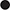, Transect A1 (the 7th Chinese National Arctic Research Expedition); 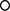, Transect A2 (the Kuroshio Extension Cruise); 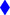, Transect B (the 10th Chinese National Arctic Research Expedition). The yellow shaded area shows the general location of the North Pacific Transition Zone.

Tintinnids were sampled at 108 stations along two major transects (Transects A and B) covering the NPTZ during three cruises (Figure 1). Along the west coast of the Pacific Ocean, Transect A consists of 51 survey stations from two cruises. Transect A1 was sampled from St. A1 to A28 during the 7th Chinese National Arctic Research Expedition aboard R.V. “*Xuelong*” from September 12 to 18, 2016. Transect A2 was surveyed from St. A51 to A29 during the Kuroshio Extension Cruise aboard R.V. “*Dongfanghong 3*” from September 4 to 12, 2019. Transect B with a total of 57 stations was sampled (from St. B1 to B57) during the 10th Chinese National Arctic Research Expedition aboard R.V. “*Xiangyanghong 10*” from September 9 to 21, 2019.

Tintinnid samples were collected during cruises with an onboard continuous underway sampling system at 5-m depth. A large volume of seawater (80 L) was gently filtered through a 10-μm mesh net. The samples (∼150 ml) in the cod end of the net were transferred into sample bottles and immediately fixed with Lugol’s solution (1% final concentration). Samples were kept in a cool, dark environment for preservation. Surface water temperature (°C) and salinity were determined using a WTW Cond 3210 SET 1 portable water quality analyzer (Xylem, Germany).

### Sample Analysis and Species Identification

In the laboratory, a subsample (25 ml or a larger volume if tintinnids were scarce) from each original sample was settled in an Utermöhl counting chamber for at least 24 h and examined using an Olympus IX 71 inverted microscope (Olympus, Tokyo, Japan) at a magnification of ×100 or ×400. At least 20 individuals (if possible) of each species were photographed and measured. Tintinnid species were identified based on lorica morphology and size according to the literature (Kofoid and Campbell, 1929; Hada, 1937, 1938; Kofoid and Campbell, 1939; Bakker and Phaff, 1976; Yoo et al., 1988; Yoo and Kim, 1990; Zhang et al., 2012; Zhang et al., 2014). As mechanical disturbance (including sample collection and fixing) can easily provoke tintinnid protoplasts to detach from the lorica (Paranjape and Gold, 1982; Gómez, 2007), intact lorica with protoplasts inside or not was counted as living cells in our study. Tintinnids were divided into neritic and oceanic species according to Dolan et al. (2013b).

### Data Processing

Tintinnid species richness in each station indicated the number of tintinnid species that appeared in this station. Abundance of each tintinnid species (*A*_i_, ind L^–1^) in each station was calculated using the following equation:

$$A_{i}=\frac{V\times N_{i}}{80v}$$

Where *V* (L^–1^) was the volume of the sample, *N*_i_ was the individual number of species *i* in the subsample, and *v* was the volume of the subsample (L^–1^). Total tintinnid abundance in each station was the sum of each tintinnid abundance appeared in this station. Occurrence frequency (OF) of each tintinnid species was calculated as the percentage of samples in which the species appeared (OF = *n* × 100/108, where *n* was the number of stations this species appeared in).

Distributional data are presented as scatter diagrams and bar charts by Grapher (Version 12, Golden Software Inc., Golden, CO, United States). Cluster analysis was performed using PRIMER (Version 5.0, PRIMER-e, Plymouth, United Kingdom) based on the abundances of different tintinnid species at each station. Group-average linkage based on the Bray–Curtis similarity matrix of the fourth root transformed from the original data was used. Correlation analysis between environmental and biological variables, and paired-sample *t*-tests to identify lorica oral diameter (LOD) differences between different communities were performed using SPSS (Version 16, SPSS Inc., IBM Corp., Armonk, NY, United States).

## Results

### General Description

**FIGURE 2:**
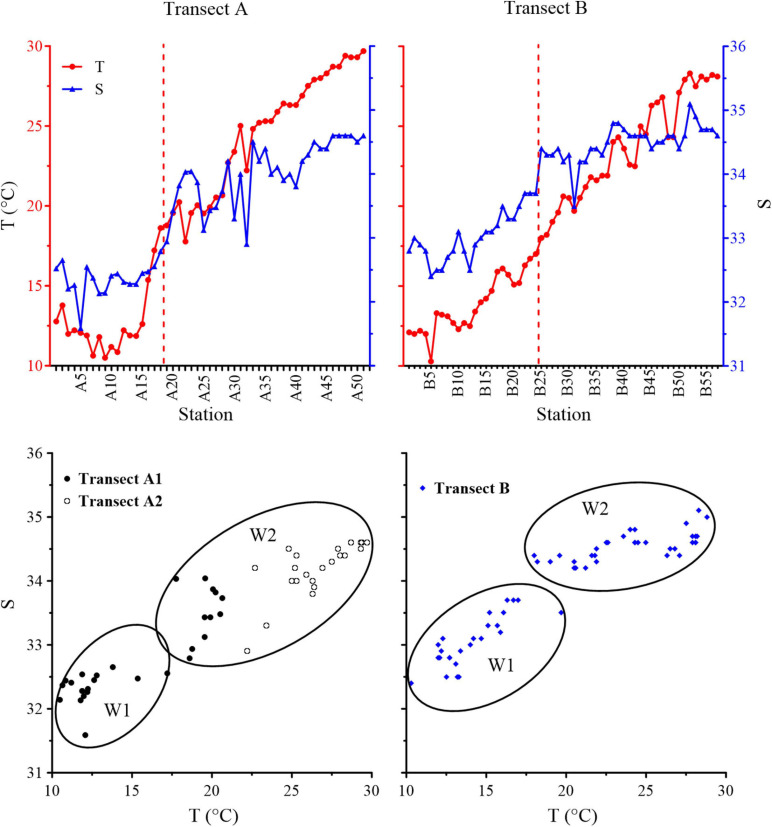
Temperature (T) and salinity (S) distributions and diagrams along Transects A and B. Red dashed lines are the locations of the subarctic Pacific water (W1) and North Pacific water (W2) divided by 17.5°C.

From north to south along the two transects, the temperature gradually increased while the salinity gradually decreased, and the range of variation was approximately the same for both transects. The variation of temperature ranged in 10.48–29.7 and 10.3–28.3°C in Transect A and Transect B, respectively. The variation range of salinity in Transect A was 31.59–34.6, and that in Transect B was 32.4–35.1. In Transect A, salinity increased linearly with temperature along the full transect. In Transect B, salinity increased linearly with temperature up to 17.5°C, above which the rate of the salinity increase with temperature decreased. For the same temperature, the corresponding salinity in Transect A is lower than that in Transect B. According to T–S diagrams, subarctic and subtropical waters were divided in two transects; 17.5°C isotherm was the boundary between subtropical and subarctic waters (Figure 2).

In total, 94 species of tintinnids (Table 1 and Supplementary Figures 1–9) were identified from the samples. The maximum abundance of different tintinnid species ranged from 0.11 ind L^–1^ (*Parundella aculeata* and *Eutintinnus haslae*) to 745.98 ind L^–1^ (*Acanthostomella norvegica*); and the average abundance ranged from 0.001 (*P. aculeata*) to 51.40 ind L^–1^ (*A. norvegica*). *Dadayiella ganymedes* had the highest OF of 60.19%, while *Undella turgida* was the least frequent species (0.96%).

**TABLE 1:** Station number of occurrence (*n*) and maximum abundance (*A*_max_, ind L^–1^) of each tintinnid species.

**Species**	***n***	***A*_max_**	**Species**	***n***	***A*_max_**	**Species**	***n***	***A*_max_**
**Oceanic species**			*Rhabdonella cornucopia*	33	1.34	*Parafavella promissa*	5	3.43
**Boreal species**			*Rhabdonella elegans*	35	2.77	*Parundella aculeata*	1	0.11
*Acanthostomella norvegica*	56	745.98	*Rhabdonella exilis*	15	1.79	*Parundella caudata*	1	0.12
*Codonellopsis frigida*	52	232.67	*Rhabdonella indica*	33	6.76	*Parundella lachmanni*	2	8.61
*Parafavella faceta*	23	8.86	*Rhabdonella sanyahensis*	27	1.33	*Petalotricha aperta*	1	0.53
*Parafavella gigantean*	22	21.48	*Steenstrupiella gracilis*	18	1.13	*Petalotricha major*	1	0.22
*Parafavella jorgenseni*	43	67.82	*Steenstrupiella robusta*	16	5.84	*Poroecus curtus*	3	0.12
*Ptychocylis obtuse*	34	284.26	**Transition zone species**			*Proplectella claparedei*	8	3.24
*Salpingella* sp. 1	41	339.70	*Undella californiensis*	10	9.16	*Proplectella ostenfeldi*	3	0.12
*Salpingella* sp. 2	12	12.08	*Undella clevei*	19	65.32	*Proplectella perpusilla*	2	0.25
*Undella* sp.	11	11.91	**Cosmopolitan species**			*Protorhabdonella striatura*	6	0.55
**Warm water type I species**			*Salpingella acuminata*	25	25.20	*Rhabdonella conica*	9	0.51
*Amphorides minor*	20	8.21	*Salpingella faurei*	45	12.08	*Rhabdonella valdestriata*	4	1.29
*Dadayiella ganymedes*	65	301.05	**Rare species**			*Salpingacantha unguiculata*	3	12.27
*Dictyocysta mitra*	20	21.28	*Acanthostomella lata*	1	0.44	*Salpingella minutissima*	1	1.38
*Eutintinnus pacificus*	42	629.55	*Amphorellopsis acuta*	3	10.27	*Undella turgida*	1	0.12
*E. tubulosus*	46	109.85	*Ascampbelliella retusa*	3	0.18	*Xystonella lanceolata*	6	0.49
*Protorhabdonella simplex*	28	23.27	*Climacocylis scalaria*	4	0.69	*Xystonella treforti*	2	0.38
*Steenstrupiella steenstrupii*	45	211.60	*Climacocylis scalaroides*	8	0.56	*Xystonellopsis brandti*	2	0.36
**Warm water type II species**			*Codonella aspera*	5	0.56	*Xystonellopsis heros*	2	0.45
*Acanthostomella minutissima*	26	3.25	*Codonellopsis contracta*	1	0.53	*Amphorellopsis* sp.	6	9.52
*Amphorides amphora*	40	11.29	*Codonellopsis meridionalis*	2	0.34	*Eutintinnus* sp.	1	1.01
*Amphorides brandti*	11	1.46	*Codonellopsis morchella*	3	7.84	**Neritic species**		
*Amphorides quadrilineata*	17	9.26	*Coxliella laciniosa*	5	0.36	*Favella panamensis*	3	13.09
*Ascampbelliella armilla*	39	12.40	*Dictyocysta polygonata*	2	0.49	*Helicostomella longa*	2	0.69
*Dictyocysta reticulata*	16	0.87	*Dictyocysta speciosa*	1	0.55	*Helicostomella subulata*	14	13.12
*Epiplocylis constricta*	13	1.47	*Epiplocylis undella*	9	0.74	*Leprotintinnus simplex*	1	0.19
*Epiplocyloides reticulata*	13	1.38	*Eutintinnus elegans*	7	0.56	*Tintinnopsis baltica*	9	9.06
*Eutintinnus apertus*	27	3.23	*Eutintinnus haslae*	1	0.11	*Tintinnopsis beroidea*	11	5.42
*Eutintinnus fraknoii*	42	31.83	*Eutintinnus macilentus*	3	0.67	*Tintinnopsis glans*	1	0.57
*Eutintinnus lusus-undae*	44	11.32	*Eutintinnus turris*	4	1.01	*Tintinnopsis kofoidi*	6	2.71
*Eutintinnus stramentus*	37	4.90	*Metacylis sanyahensis*	2	0.70	*Tintinnopsis meunieri*	1	0.25
*Protorhabdonella curta*	48	4.84	*Parafavella denticulata*	3	0.37	*Tintinnopsis spiralis*	3	20.00
*Rhabdonella amor*	17	2.49	*Parafavella pacifica*	3	0.77	*Tintinnopsis* sp.	3	1.38

Most of the identified tintinnid species (83 species) were oceanic; only 11 neritic species (Table 1 and Supplementary Figure 1) were observed. In general, oceanic species dominated the total tintinnid abundance. With the maximum abundance of total neritic species of 30.20 ind L^–1^ at St. A1 (Supplementary Figure 10), neritic species accounted for less than 5% of the total tintinnid abundance except at six stations (St. A1, A2, A3, A5, A19, and B5, ∼5.67–36.50%) (Supplementary Figure 10). Most of these neritic species occurred at less than 10 stations, except for *Tintinnopsis beroidea* and *Helicostomella subulata* (Table 1). Neritic species mainly appeared in the northern coastal part of Transect A. In Transect B, only two neritic species appeared at four stations with extremely low abundance (Supplementary Figure 10).

### Distribution Patterns of Common Oceanic Species

Among the 83 oceanic tintinnid species, 41 species occurred at more than 10 stations (OFs > 9%). These species were defined as common oceanic species. The remaining 42 oceanic species with OFs < 9% were defined as rare species (Table 1). According to their abundance distribution patterns along the temperature profiles in the transects, common oceanic species could be further divided into five groups: boreal species, warm water type I species, warm water type II species, transition zone species, and cosmopolitan species.

**FIGURE 3:**
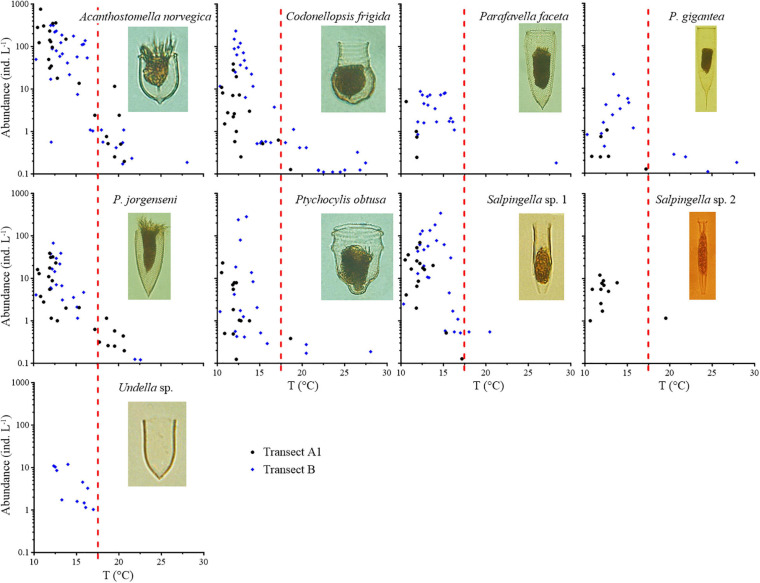
Abundance variation of boreal species along the temperature (T) gradient. Red dashed lines show the boundary between the subarctic Pacific water and North Pacific water, i.e., the location of 17.5°C.

Nine boreal species (Figure 3, Supplementary Figure 2, and Table 1) were characterized by a decrease in abundance with increasing temperature. Among them, *A. norvegica*, *Ptychocylis obtusa*, *Codonellopsis frigida*, and *Salpingella* sp. 1 had maximum abundances greater than 100 ind L^–1^ (Figure 3). Four boreal species, *A. norvegica*, *C. frigida*, *Parafavella gigantea*, and *P. obtusa*, extended far southward into warmer waters (27.5°C); while *Parafavella faceta*, *Undella* sp., *Salpingella* sp. 1, and *Salpingella* sp. 2 were mainly distributed in waters cooler than 17.5°C. Generally, boreal species extended farther south in Transect B than in Transect A.

**FIGURE 4:**
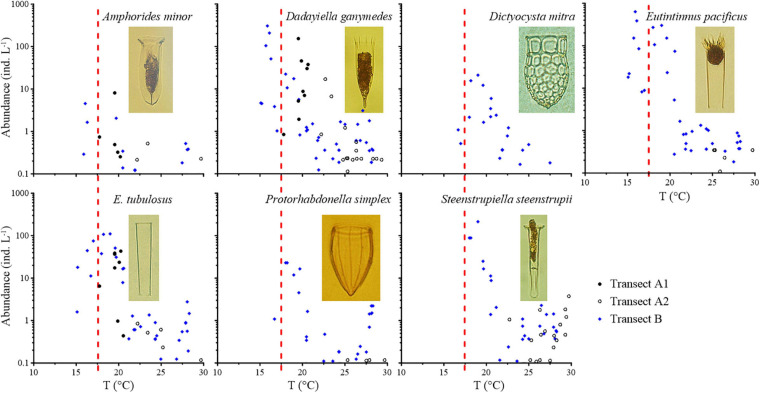
Abundance variation of warm water type I species along the temperature (T) gradient. Red dashed lines show the boundary between the subarctic Pacific water and North Pacific water, i.e., the location of 17.5°C.

Seven warm water type I species (Figure 4, Supplementary Figure 3, and Table 1) were found in waters with temperatures above 15°C. They were the most abundant from 15 to 20°C. When the temperature exceeded 20°C, the abundance of warm water type I species decreased. Four species (*D. ganymedes*, *Eutintinnus pacificus*, *Steenstrupiella steenstrupii*, and *Eutintinnus tubulosus*) had maximum abundances higher than 100 ind L^–1^ (Figure 4). Warm water type I species extended farther north in Transect B than in Transect A.

**FIGURE 5:**
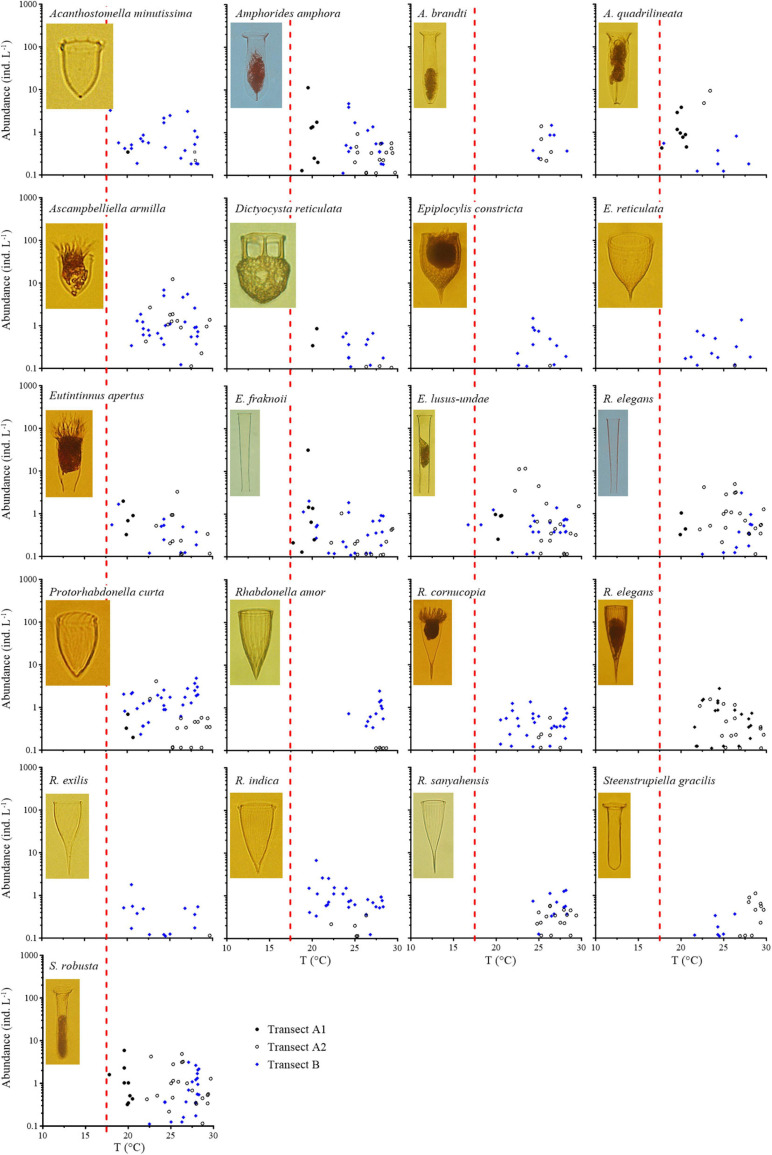
Abundance variation in warm water type II species along the temperature (T) gradient. Red dashed lines show the boundary between the subarctic Pacific water and North Pacific water, i.e., the location of 17.5°C.

Twenty-one species (Figure 5, Supplementary Figure 4, and Table 1) were classified as warm water type II species. Maximum abundances of these species were less than 13 ind L^–1^, except *Eutintinnus fraknoii*. All these species mainly occurred in >17.5°C waters. The lower temperature limits of the distributions of warm water type II species also varied (*Rhabdonella amor*, *Amphorides brandti*, and *Rhabdonella sanyahensis* 24.3°C; *Rhabdonella elegans* and *Steenstrupiella gracilis* 21.6°C; *Ascampbelliella armilla* and *Epiplocyloides reticulata* 20.5°C; *Rhabdonella cornucopia* 19.7°C; *Protorhabdonella curta*, *Rhabdonella exilis*, and *Rhabdonella indica* 19.6°C; *Dictyocysta reticulata* 19.5°C; and *Eutintinnus apertus* 18.2°C). Only *Eutintinnus lusus-undae* occurred once in waters colder than 17.5°C.

**FIGURE 6:**
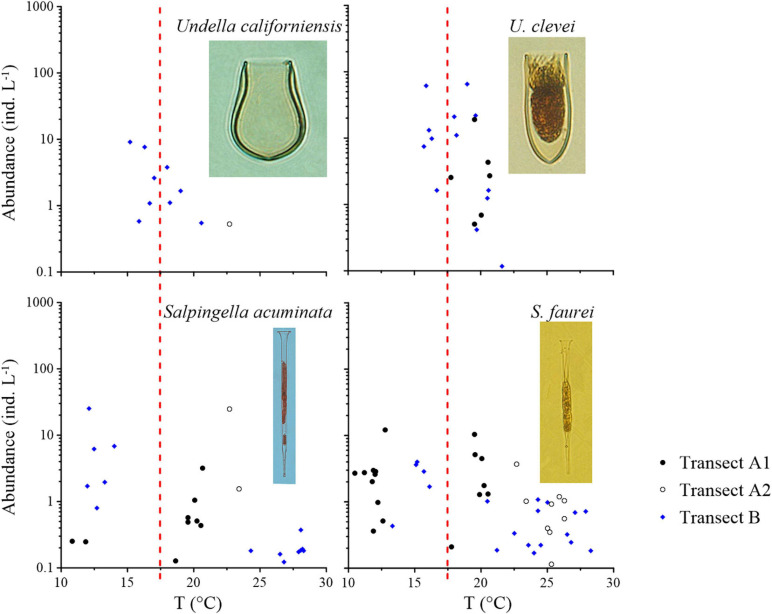
Abundance variation of transition zone species (*Undella californiensis* and *Undella clevei*) and cosmopolitan species (*Salpingella acuminata* and *Salpingella faurei*) along the temperature (T) gradient of the two transects. Red dashed lines show the boundary between the subarctic Pacific water and North Pacific water, i.e., the location of 17.5°C.

*Undella clevei* and *Undella californiensis* mainly occurred in the temperature range of 15–20°C with occasional appearances at >20°C waters (Figure 6, Supplementary Figure 5, and Table 1). They were considered as transition zone species. The maximum abundance was 65.32 ind L^–1^ for *U. clevei* and 9.16 ind L^–1^ for *U. californiensis*. Generally, transition zone species extended farther north in Transect B than in Transect A. Two cosmopolitan species (*Salpingella acuminata* and *Salpingella faurei*) (Supplementary Figure 6 and Table 1) showed no obvious distribution preference (Figure 6).

### Abundance and Species Richness of Oceanic Tintinnids

**FIGURE 7:**
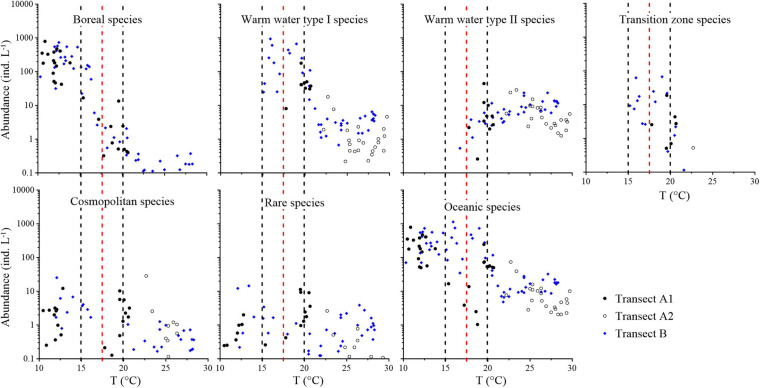
Abundance variation of different tintinnid groups and total oceanic species along the temperature (T) gradient. Red dashed lines show the boundary between the subarctic Pacific water and North Pacific water, i.e., the location of 17.5°C. Black dashed lines show the location of 15 and 20°C.

With the exception of cosmopolitan species, a distinct occurrence pattern was observed between the total abundance of different groups and temperature (Figure 7). Abundance of total boreal species decreased southward along the two transects; the abundance was much higher in Transect A than Transect B (Supplementary Figure 11). The boreal species group had a constant high abundance (30–793.40 ind L^–1^) when water temperatures were <15°C, decreased when water temperatures ranged in 15–20°C, and remained low at <3.00 ind L^–1^ with water temperatures >20°C (Figure 7). Correlation analysis showed that the relationships between abundances of all the boreal species and temperature were strongly significant negative (*p* < 0.01), while most species showed strongly significant negative correlations with salinity (*p* < 0.01; Supplementary Table 1).

Warm water type I species occurred in waters >15°C. Their abundance was higher at middle region of Transect A and north region of Transect B where temperature ranged in 15–20°C (8.13–930.90 ind L^–1^) and decreased to 0.22–37.06 ind L^–1^ in >20°C waters (Figure 7 and Supplementary Figure 11). Five of the seven warm water type I species showed significant positive correlations with temperature (*p* < 0.05), while six of them showed strongly significant positive correlations with salinity (*p* < 0.01; Supplementary Table 1). Warm water type II species abundance was constant in south regions of the two transects with >20°C waters (1.48–28.30 ind L^–1^) and decreased at northward in <20°C waters (Supplementary Figure 11); these species almost disappeared in waters cooler than 17.5°C (Figure 7). All the warm water type II species showed strongly significant positive correlations with temperature and salinity (*p* < 0.01), except *Amphorides quadrilineata*, which showed no significant relationship with temperature and salinity (Supplementary Table 1).

Higher abundance region of transition zone species was similar with warm water type I species (Supplementary Figure 11). This group had higher abundance (0.41–67.00 ind L^–1^) at 15–20°C waters and lower abundance (0.12–2.72 ind L^–1^) at >20°C waters. They disappeared in waters with temperature <15°C. Cosmopolitan species and rare species occurred along the two transects in the full temperature range with low abundance and no temperature preference (Figure 7 and Supplementary Figure 11). In summary, total abundance of oceanic tintinnids ranged from 1.04 to 1,146.39 ind L^–1^, with constant high abundance at temperatures of 10–20°C and low abundance at temperatures >20°C. Correlation analysis showed that both transition zone species and cosmopolitan species showed no significant relationships with temperature or salinity (Supplementary Table 1).

**FIGURE 8:**
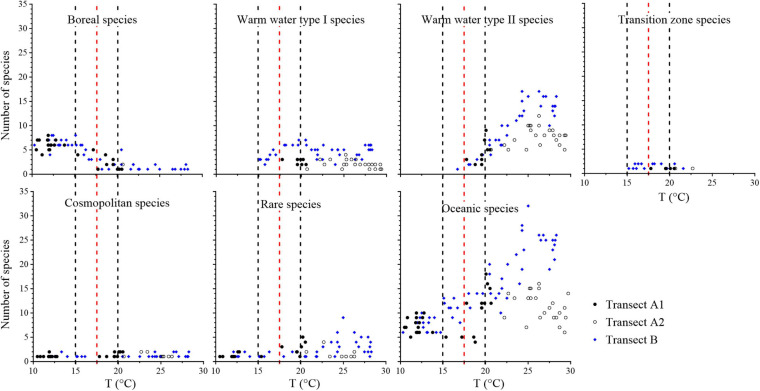
Variations in the number of species in different groups and total oceanic species along the temperature (T) gradient. Red dashed lines show the boundary between the subarctic Pacific water and North Pacific water, i.e., the location of 17.5°C. Black dashed lines show the location of 15 and 20°C.

Oceanic tintinnid species richness ranged from 4 to 18 along Transect A, with no clear trend. In Transect B, oceanic tintinnids species richness increased from north to south with a range of 6–32 (Figure 8). The number of boreal species decreased with increasing temperature and remained at low values from 15 to 20°C. The number of warm water type II species was higher in warm waters but decreased when the temperature was below 25°C; these species almost disappeared when the temperature was under 15°C. The number of rare species was lower than 9 at all stations. Boreal species extended farther southward in Transect B than in Transect A and vice versa in the case of warm water type I species and transition zone species (Figure 8).

### Oceanic Tintinnid Community

**FIGURE 9:**
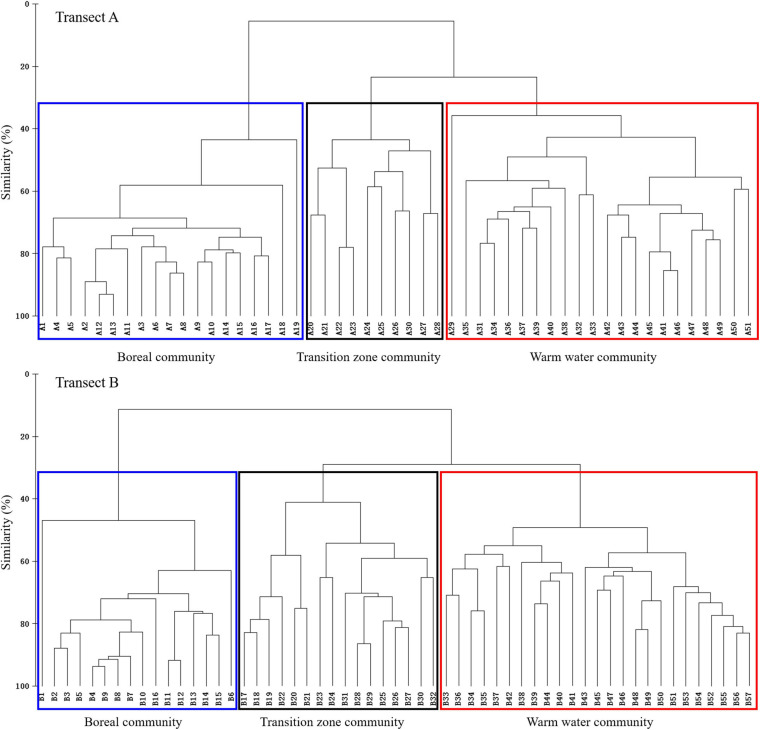
Boreal, transition zone, and warm water communities in Transects A and B as revealed by cluster analysis.

**FIGURE 10:**
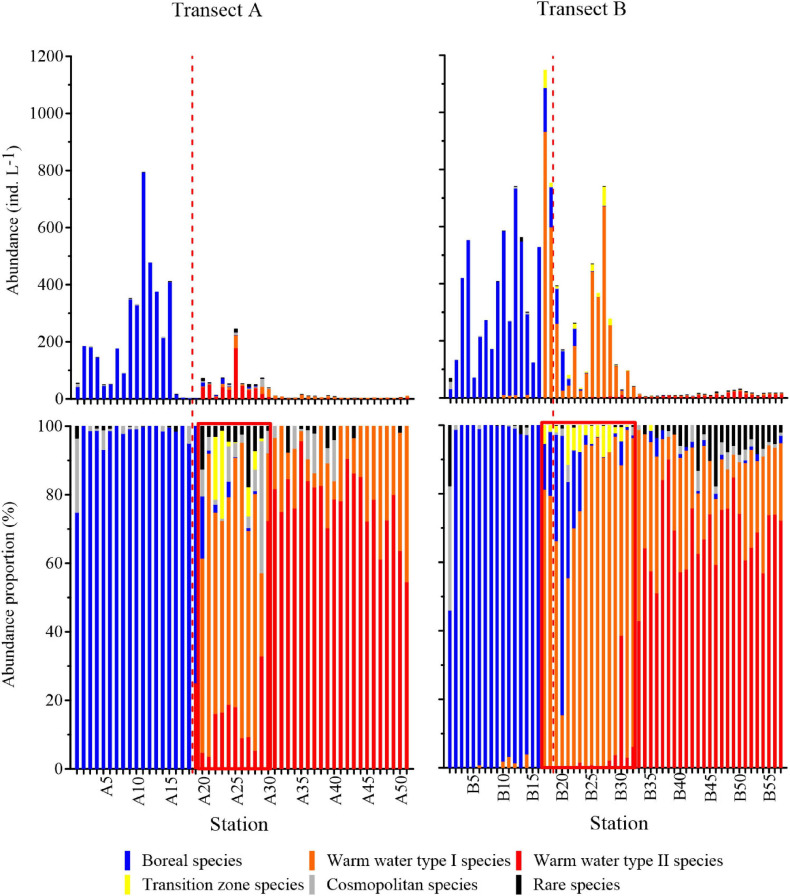
Abundances and abundance proportions of oceanic tintinnids in different groups. Red dashed lines are the locations of the subarctic Pacific water and North Pacific water divided by 17.5°C. The red box is the transition zone community. Boreal and warm water communities are located to the left and right of the transition zone community, respectively.

Based on cluster analysis using oceanic species data, three tintinnid communities were identified in the surface waters of the two transects (Figure 9), with St. A20–A30 (except St. A29) and St. B17–B32 containing the transition zone community. The transition zone community expanded in waters with temperatures <17.5°C in Transect B (north of St. B25). In contrast, it was restricted at temperatures >17.5°C (south of St. A18) in Transect A. The temperature ranges of the transition zone community were 17.76–23.40 and 15.1–20.6°C, while the salinity ranges were 33.30–34.04 and 33.2–34.4 in Transects A and B, respectively. The boreal community and warm water community were located to the north and south of the transition zone community, respectively. Five common oceanic tintinnid groups and rare species differed in their contributions to different communities (Figure 9). Boreal species, warm water type I species, and warm water type II species dominated (>50% abundance proportion) in the boreal community, transition zone community, and warm water community, respectively (Figure 10).

**FIGURE 11:**
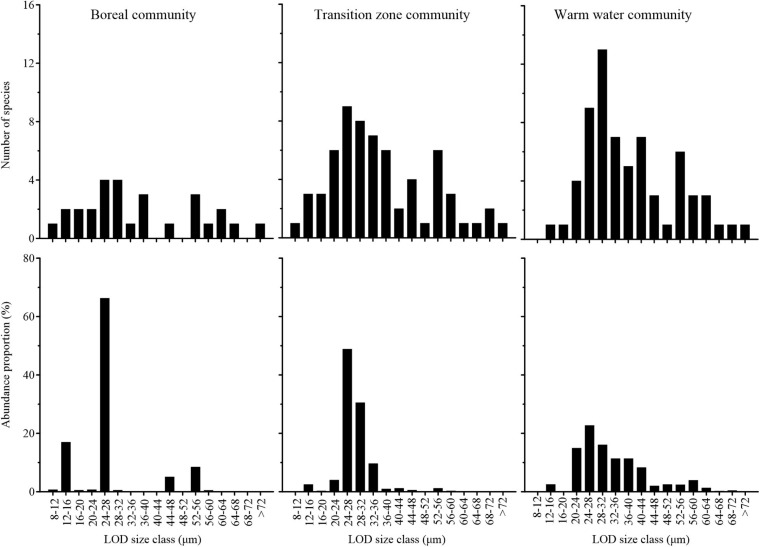
Number of species and abundance proportion of each lorica oral diameter (LOD) size class in three tintinnid communities.

The number of species and abundance proportion of each LOD size class in the different tintinnid communities are shown in Figure 11. There were 28 tintinnid species with 14 LOD size classes in the boreal community. The number of species in each LOD size class ranged in 1–6. In the transition community, 61 tintinnid species in 17 LOD size classes were found; the number of species was less than 10 in each LOD size class. Sixty-six tintinnid species with 16 LOD size classes appeared in the warm water community, with the number of species in each LOD size class ranged in 1–13. In the boreal community, the 24–28 μm LOD size class was in overwhelming superiority and occupied 66.26% of total boreal community abundance. In the transition zone community, the 24–28 μm LOD size class contributed 48.85% to total community abundance, followed by 28–32 μm LOD size class that contributed 30.53% to total community abundance. In the warm water community, abundance proportions of each LOD size class were more evenly distributed. The 24–28 μm LOD size class still was the first contributor with the abundance proportion of 22.72%, followed by 24–28 μm (16.05%), 16–20 μm (14.93%), 36–40 μm (11.36%), and 32–36 μm (11.35%) (Figure 11).

Paired-sample *t*-tests based on number of species in each LOD size class showed that the differences between the boreal community and the other two communities were significant (*p* < 0.05), while there was no significant difference between the transition zone community and warm water community (*p* > 0.05; Supplementary Table 2).

## Discussion

### Transition Zone Species

Two transition zone species were noted in this study. Those two species occurred at regions with moderate temperature and disappeared at regions with higher or colder temperature. This may the reason that correlation analysis did not show significant relationships between transition zone species and temperature.

*Undella californiensis* was previously found on the mid-latitude coast of Japan (Balech, 1975), in the California Current (Point Conception) (Kofoid and Campbell, 1929; Balech, 1975) and in the eastern tropical Pacific (Kofoid and Campbell, 1939). In a short transect at 140°E, 30–35°N (Figure 1), Gómez (2007) reported a very low abundance of this species in May 2002, although the exact location of *U. californiensis* observed in that transect was not specified. It is reasonable to assume that *U. californiensis* occurs in the northern part of the transect, i.e., north of the Kuroshio axis, due to the nature of transition zone species. All the information supports that *U. californiensis* is endemic to the NPTZ.

*Undella clevei* was also found in transition zone waters such as the Tsushima Strait (Kim et al., 2012), Transect 140°E, 30–35°N (Gómez, 2007), and the California Current (Dolan et al., 2013a). However, its occurrence was also reported in the Mediterranean Sea (Dolan, 2017; Njire et al., 2019), the western tropical Pacific (Kieu et al., 2017; Li et al., 2018), the central equatorial Pacific (Gómez, 2007), and the South Pacific (Dolan et al., 2006). Therefore, although we classified *U. clevei* as a transition zone species, it might be a widespread warm water species.

Only two transition zone species were found in this study. This number was very small compared with the large species richness. However, it was consistent with the small number of other transition zone plankton groups in previous studies. There were only seven phytoplankton species (*Chaetoceros peruvianus*, *Rhizosolenia hebetata*, *Pseudoeunotia doliolus*, *Actinocyclus curvatulus*, *Hemidiscus cuneiformis*, *Coscinodiscus eccentricus*, and *Coscinodiscus stellaris*) (Venrick, 1971), one chatognath species (*Sagitta scrippsae*), three copepod species (*Eucalanus elongates hyalinus*, *Eucalanus bungii californicus*, and *Clausocalanus pergens*), four euphausiid species (*Thysanopoda acutifrons*, *Thysanoessa gregaria*, *Euphausia gibboides*, and *Nematoscelis difficilismegalops*), and two pteropod species (*Corolla pacifica* and *Clio balantium*) endemic to the NPTZ (McGowan, 1971).

### Warm Water Type I Species

In the present study, *Amphorides minor*, *D. ganymedes*, *Dictyocysta mitra*, *E. pacificus*, *E. tubulosus*, *Protorhabdonella simplex*, and *S. steenstrupii* were considered warm water type I species. Most of them have been widely documented in both neritic and oceanic waters. *D. ganymedes* and *S. steenstrupii* are well-known warm water species in the Pacific, Atlantic, and Indian Oceans (Dolan et al., 2013b). *A. minor* was found in coastal (Jyothibabu et al., 2008; Saab et al., 2012; Li et al., 2016b, 2019; Jung et al., 2017) and warm oceanic waters (Ota and Taniguchi, 2003; Kim et al., 2012; Li et al., 2018). *E. pacificus* occurs in warm waters (Kim et al., 2012; Li et al., 2016a, 2018), continental shelf waters (Li et al., 2016b; Yu et al., 2016), and the region of the Kuroshio and Oyashio Currents (Gómez, 2007). *E. tubulosus* was found in neritic waters (Li et al., 2020), continental shelf waters (Li et al., 2016b; Yu et al., 2016), and open seas (Li et al., 2018). *P. simplex* was present in the western tropical Pacific (Li et al., 2018; Wang et al., 2019), East China Sea (Li et al., 2016b), and Mediterranean Sea (Njire et al., 2019). *D. mitra* was mainly reported in relatively warm waters (Modigh et al., 2003; Dolan et al., 2006, 2013a; Gómez, 2007; Jyothibabu et al., 2008; Kim et al., 2012), but also occurred in the Southern Ocean (Thompson, 2004; Dolan et al., 2012).

Previous studies on the biogeographical ranges of tintinnids were mainly based on the presence/absence of individual species and lacked details of abundance changes. Our study provides details of the abundance changes in seven warm water type I species in the vicinity of the NPTZ. The results indicate that warm water type I species are species with broad distributions but maximum abundances in the transition zone, which is a feature of the transition zone in addition to transition species (Venrick, 1971).

The north–south extent of warm water type I species is asymmetric. Their abundance is higher in the NPTZ and decreases abruptly when the temperature falls below 15°C. Therefore, the occurrence/disappearance of this group can be used as an indicator of the northern boundary of the NPTZ. Moreover, warm water type I species could also be used to denote the northward expansion of plankton due to global warming (Beaugrand et al., 2009).

### Tintinnid Community Variation Across the North Pacific Transition Zone

The NPTZ is the dividing line between warm water (subtropical) and boreal (subarctic) planktons (Roden, 1991; Yamaguchi et al., 2017). However, it has different blocking effects on the warm water and boreal communities. From subarctic water to subtropical water in the North Pacific, tintinnid community varied from the boreal community to the transition zone community and to the warm water community. The boreal tintinnid community extended southward farther than the warm water community extended northward. South of the NPTZ, when the temperature was below 23°C, the species number of warm water type II species began to decline. To our knowledge, no similar results were found in other plankton groups. Due to the decrease in the number of species of both boreal species and warm water type I species, the total species richness in the transition zone community was generally higher than that in the boreal community but lower than that in the warm water community. Similar studies have been conducted in the Southern Ocean and East China Sea. The species richness in the polar front was higher than that in the subantarctic and Antarctic waters on either side of it (Liang et al., 2020). In contrast, both neritic and oceanic communities had higher species richness than transitional communities in the East China Sea (Li et al., 2016b).

Lorica oral diameter is an important index of tintinnid community and closely related to most of tintinnid ecological parameters (including growth rate and size of prey). The size of the largest prey is about 45% of the LOD, and the size of the preferred prey (removed at maximum rates) is about 25% of the LOD (Dolan, 2010). We found that the LOD size class 24–28 μm had the greatest abundance proportion in all three communities, but the dominance of this size class decreased and the abundance proportions of each size class become more even from the boreal community to the transition zone community and then to the warm water community. This indicated that the size of tintinnid main prey did not change from subarctic to subtropical, but the dominance of this size prey decreased and abundance proportion of other size preys increased from subarctic to subtropical waters. Although the number of tintinnid species was low than the warm water community, the number of LOD size classes was the highest in the transition zone community. This may indicate that the diversity of prey was much higher in the NPTZ than the adjacent waters.

Wang et al. (2020) compared the number of species and abundance proportions of LOD size classes in warm water (western tropical Pacific), boreal (Bering Sea), and Arctic tintinnid communities. The LOD size class characteristics of the warm water and boreal communities in our study were similar to those of Wang et al. (2020). The spectrum of LOD size class characteristics of the transition zone community fit well within the spectrum of LOD size classes from warm water to the Arctic.

McGowan (1971) was the only study to examine the variation in abundance of subarctic, NPTZ, and subtropical plankton groups along 155°W. The abundance of transition zone group was comparable with that of subtropical group in that research. In our study, the abundance of transition zone tintinnids was lower, while the abundance of warm water type I species was higher than that of warm water type II species. The abundance variation patterns of boreal, transition zone, and warm water type II species in our study were similar to those of McGowan (1971).

### Range of Tintinnid Transition Zone Community

Transition zone species and warm water type I species had high abundances at 15–20°C and disappeared abruptly below 15°C. When the temperature was between 15 and 20°C, the abundance of boreal species decreased with increasing temperature and remained at a lower level until boreal species disappeared at 27°C. Warm water type II species were more abundant at >20°C waters and less abundant at 15–20°C waters, rarely extending to <15°C waters. Cluster analysis also showed that tintinnid transition zone community mainly appeared at regions with temperature ranged in 15–20°C. Therefore, we chose 15–20°C as the basis for delineating the transition zone community in this study. The northern boundary of tintinnid transition zone community was in accordance with Ekman (1953), who considered 15°C as the temperature boundary of warm water and boreal epiplanktonic fauna in summer.

The latitudinal width of the transition zone community in our study is 4° latitude (41°–45°N in Transect A and 40°–44°N in Transect B), which is consistent with the physical width of Uda (1963) (approximately 2° to 4° latitude), but less than the width of the of McGowan (1971) (7°–8° latitude). In the present research, 15°C was located near 45°N, which is also the northern boundary of the transition zone (40°–45°N) (Reid et al., 1978). During different months of the year, the water temperature and the latitudinal position of the NPTZ may vary greatly (Church et al., 2008; Howell et al., 2012; Follett et al., 2021; Navarra and Di Lorenzo, 2021). This may cause the northward or southward move of the transition zone community in different months.

### Difference Among Transects

In the present study, 11 neritic tintinnids were identified in Transect A, while only two neritic species were identified in Transect B. This may because the stations in Transect A were much closer to the shore and were influenced much stronger by nearshore waters than those in Transect B.

The southern parts of Transect A and Transect B differed in species richness, although they had similar latitude ranges. St. A51 and St. B57 were located very close to each other. However, the sampling dates at these two stations were 16 days apart. Surface temperature at St. A51 (29.7°C) was higher than that at St. B57 (28.1°C), but the salinity was the same (34.6). Rapid changes in water masses might be responsible for the difference in species richness between these two stations.

In our study, both warm water type I species and transition zone species extended from warm water to 15 and 17.5°C in Transects B and A, respectively. Boreal species extended from cold water to 28 and 20°C in Transects B and A, respectively. This implies that both boreal and warm water type I species (and transition zone species) invaded each other to some extent. This difference between the two transects might be due to different sampling times. Another possibility is that the transition zone is farther south in Transect A (Backus, 1986; Brodeur et al., 1996). The distance between the warm water type I species and boreal species distribution areas was greater in Transect A than in Transect B, which might be due to the larger distance between the Kuroshio and Oyashio Currents in Transect A (Brodeur et al., 1996). Lower salinity in Transect A due to its proximity to land might affect the spatial extent of these oceanic species (Li et al., 2016b).

The transition zone species *U. californiensis* was not found in a previous study (Li et al., 2016a) along a transect from the Japan Sea through the Soya Strait to the North Pacific along the east coast of Kamchatka. In that study, three warm water type I species (*E. pacificus*, *D. ganymedes*, and *A. minor*) were present in only the Japan Sea, but their maximum abundances were much lower than those in the present study. The other four warm water type I species (*D. mitra*, *E. tubulosus*, *P. simplex*, and *S. steenstrupii*) that were found in our study were not present in Li et al. (2016a). The lower abundance of these species might be explained by the higher temperature (19–25°C) along the Japan Sea transect (Li et al., 2016a). Therefore, the transition zone was absent in the Japan Sea transect (Li et al., 2016a).

## Conclusion

In this study, we investigated tintinnid community variations along two transects across the NPTZ. More neritic tintinnid species occurred in Transect A, which is along the west coast of the North Pacific, compared with Transect B. Boreal, warm water type I, warm water type II, transition zone, and cosmopolitan tintinnid groups were identified based on the abundance variation of each tintinnid species along with temperature. *U. clevei* and *U. californiensis* were transition zone species in the NPTZ, while *U. californiensis* was restricted in the NPTZ and adjacent region. Boreal, transition zone, and warm water tintinnid communities appeared successively along the two transects from subarctic to subtropical waters. Warm water type II species was the main contributor, while the contribution of transition zone species was relatively small (<4.75%) in the transition zone community. In the transition zone community, tintinnid species richness was higher than the boreal community but lower than the warm water community. To our knowledge, this is the first report identifying the existence of tintinnid transition zone species and the transition zone community. In the NPTZ, the strong interactions between the Kuroshio and Oyashio Currents generate unique physical features. Therefore, future research is needed in order to describe the detail characteristics of tintinnid community in the NPTZ.

## Data Availability Statement

The original contributions presented in the study are included in the article/Supplementary Material, further inquiries can be directed to the corresponding authors.

## Author Contributions

HL, JX, and WZ field sampling, tintinnid taxonomy and counting, data analysis, and writing–original draft. CW tintinnid taxonomy and counting. ZC and GG conceptualization and cruise support. YZ conceptualization and writing–original draft. WZ conceptualization. All authors contributed to the article and approved the submitted version.

## Conflict of Interest

The authors declare that the research was conducted in the absence of any commercial or financial relationships that could be construed as a potential conflict of interest.

## Publisher’s Note

All claims expressed in this article are solely those of the authors and do not necessarily represent those of their affiliated organizations, or those of the publisher, the editors and the reviewers. Any product that may be evaluated in this article, or claim that may be made by its manufacturer, is not guaranteed or endorsed by the publisher.

## Supplementary Material

Supplementary Figure 1 Photographs of neritic species observed in this study. **(A)** *Tintinnopsis beroidea*; **(B)** *Tintinnopsis* sp.; **(C)** *T. glans*; **(D)** *T. baltica*; **(E)** *T. spiralis*; **(F)** *T. meunieri*; **(G)** *T. kofoidi*; **(H)** *Leprotintinnus simplex*; **(I)** *Helicostomella longa*; **(J)** *H. subulata*; and **(K)** *Favella panamensis*.

Supplementary Figure 2 Photographs of boreal species observed in this study. **(A)** *Undella* sp.; **(B)** *Acanthostomella norvegica*; **(C)** *Salpingella* sp. 1; **(D)** *Codonellopsis frigida*; **(E)** *Ptychocylis obtusa*; **(F)** *Salpingella* sp. 2; **(G)** *Parafavella jorgenseni*; **(H)** *P. faceta*; and **(I)** *P. gigantea*.

Supplementary Figure 3 Photographs of warm water type I species observed in this study. **(A)** *Amphorides minor*; **(B)** *Eutintinnus pacificus*; **(C)** *E. tubulosus*; **(D)** *Dadayiella ganymedes*; **(E)** *Steenstrupiella steenstrupii*; **(F)** *Dictyocysta mitra*; and **(G)** *Protorhabdonella simplex*.

Supplementary Figure 4 Photographs of warm water type II species observed in this study. **(A)** *Acanthostomella minutissima*; **(B)** *Ascampbelliella armilla*; **(C)** *Epiplocylis constricta*; **(D)** *Rhabdonella amor*; **(E)** *R. sanyahensis*; **(F)** *R. cornucopia*; **(G)** *R. elegans*; **(H)** *R. indica*; **(I)** *R. exilis*; **(J)** *Dictyocysta reticulata*; **(K)** *Epiplocyloides reticulata*; **(L)** *Protorhabdonella curta*; **(M)** *Steenstrupiella gracilis*; **(N)** *S. robusta*; **(O)** *Amphorides amphora*; **(P)** *A. quadrilineata*; **(Q)** *A brandti*; **(R)** *Eutintinnus apertus*; **(S)** *E. stramentus*; **(T)** *E. lusus-undae*; and **(U)** *E. fraknoii*.

Supplementary Figure 5 Photographs of transition zone species observed in this study. **(A)** *Undella californiensis*; **(B)** *U. clevei*.

Supplementary Figure 6 Photographs of cosmopolitan species observed in this study. **(A)** *Salpingella faureia*; **(B)** *S. cuminata*.

Supplementary Figure 7 Photographs of rare oceanic species observed in this study-part 1. **(A)** *Acanthostomella lata*; **(B)** *Ascampbelliella retusa*; **(C)** *Codonellopsis morchella*; **(D)** *C. contracta*; **(E)** *Metacylis sanyahensis*; **(F)** *Undella turgida*; **(G)** *Eutintinnus haslae*; **(H)** *E. macilentus*; **(I)** *Amphorellopsis* sp.; **(J)** *Dictyocysta polygonata*; **(K)** *D. speciosa*; **(L)** *Poroecus curtus*; **(M)** *Proplectell. perpusilla*; **(N)** *P. ostenfeldi*; **(O)** *P. claparedei*; **(P)** *Coxliella laciniosa*; **(Q)** *Petalotricha aperta*; **(R)** *P. major*; **(S)** *Parundella lachmanni*; **(T)** *P. aculeata*; **(U)** *P. caudata*; **(V)** *Epiplocylis undella*; and **(W)** *Protorhabdonella striatura*.

Supplementary Figure 8 Photographs of rare oceanic species observed in this study-part 2. **(A)** *Codonella aspera*; **(B)** *Eutintinnus elegans*; **(C)** *Eutintinnus* sp.; **(D)** *E. turris*; **(E)** *C. scalaroides*; **(F)** *Climacocylis scalaria*; **(G)** *Parafavella denticulata*; **(H)** *P. pacifica*; **(I)** *Amphorellopsis acuta*; **(J)** *Xystonellopsis brandti*; **(K)** *Xystonella lanceolata*; **(L)** *Codonellopsis meridionalis*; **(M)** *Salpingella minutissima*; and **(N)** *Salpingacantha unguiculata*.

Supplementary Figure 9 Photographs of rare oceanic species observed in this study-part 3. **(A)** *Rhabdonella valdestriata*; **(B)** *R. conica*; **(C)** *Xystonella treforti*; **(D)** *Parafavella promissa*; and **(E)** *Xystonellopsis heros*.

Supplementary Figure 10 Abundance and abundance proportion of neritic and oceanic species at each station.

Supplementary Figure 11 Abundance variation of each tintinnid oceanic group in two transects.

Supplementary Table 1 Spearman’s rank correlation coefficient between abundance of each tintinnid species and environmental factors.

Supplementary Table 2 Result of paired-sample *t* tests based on number of species in each LOD size class in different communities.
