# Supplementary figures and images for: Summertime Tintinnid Community in the Surface Waters Across the North Pacific Transition Zone
Source: Front Microbiol. 2021 Aug 11;12:697801. doi: 10.3389/fmicb.2021.697801 (PMC8386027; doi:10.3389/fmicb.2021.697801)

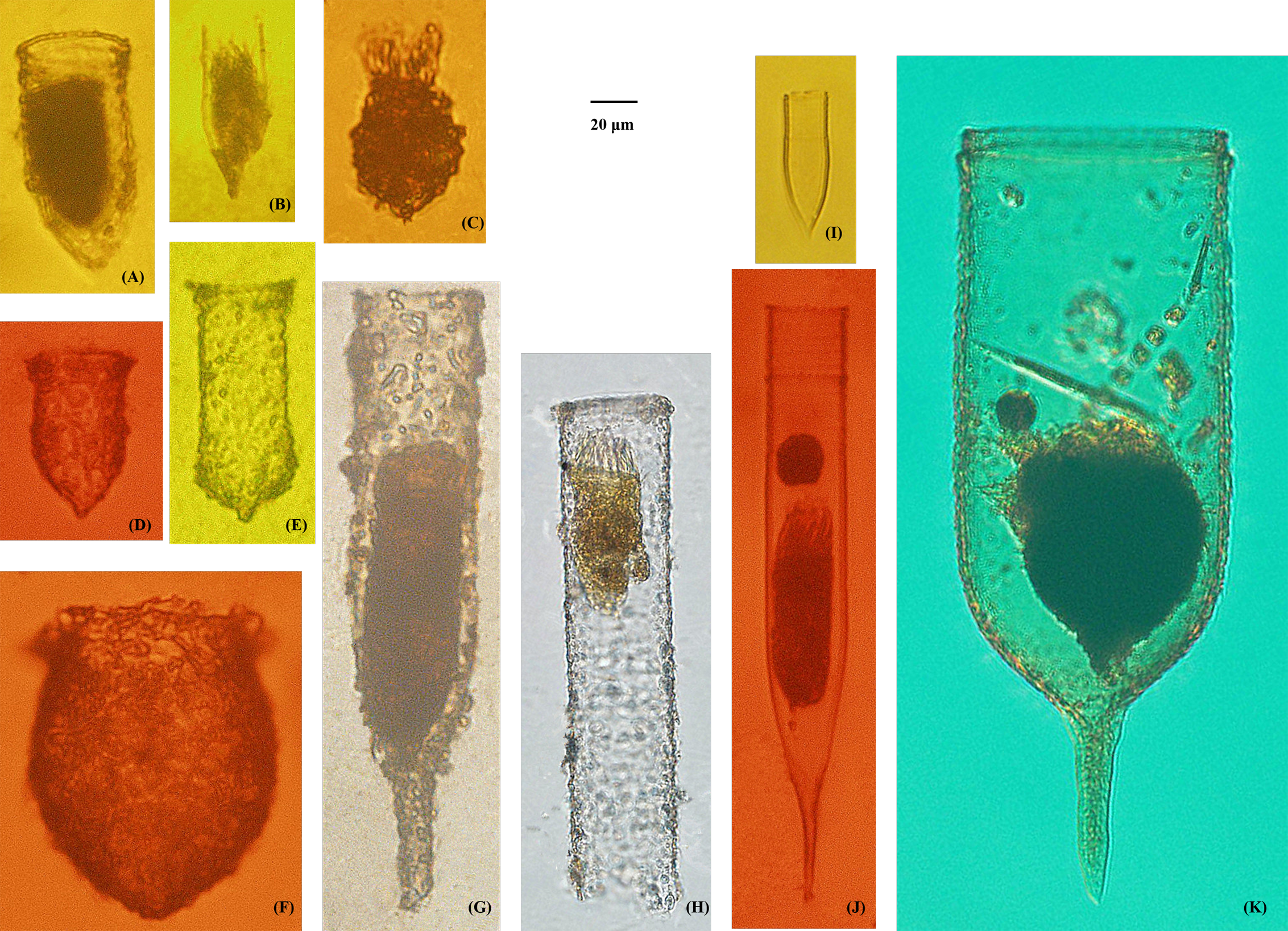

Supplement: Supplementary Figure 1 — Photographs of neritic species observed in this study. (A) Tintinnopsis beroidea; (B) Tintinnopsis sp.; (C) T. glans; (D) T. baltica; (E) T. spiralis; (F) T. meunieri; (G) T. kofoidi; (H) Leprotintinnus simplex; (I) Helicostomella longa; (J) H. subulata; and (K) Favella panamensis. [file Image_1.TIF]

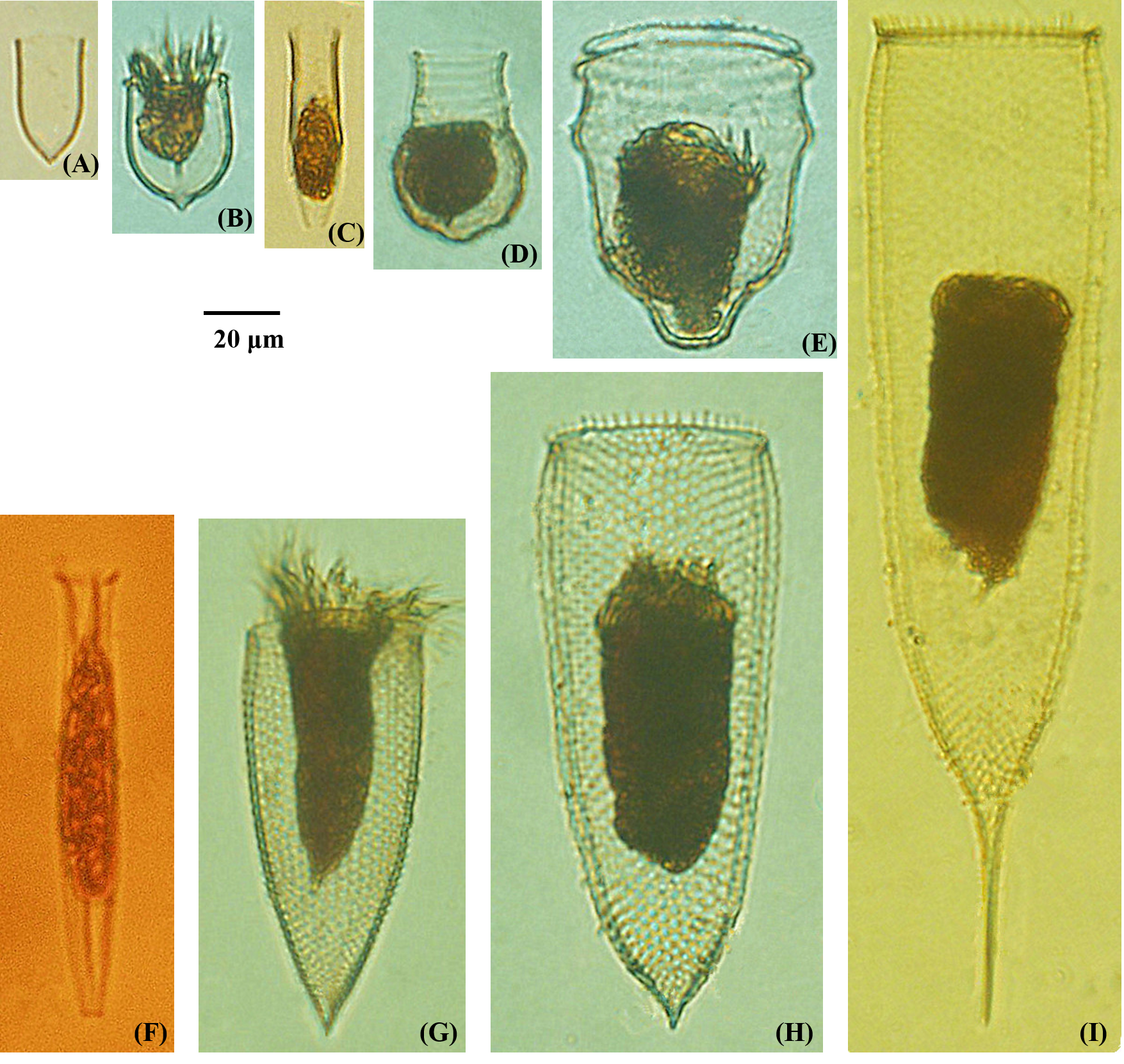

Supplement: Supplementary Figure 2 — Photographs of boreal species observed in this study. (A) Undella sp.; (B) Acanthostomella norvegica; (C) Salpingella sp. 1; (D) Codonellopsis frigida; (E) Ptychocylis obtusa; (F) Salpingella sp. 2; (G) Parafavella jorgenseni; (H) P. faceta; and (I) P. gigantea. [file Image_2.TIF]

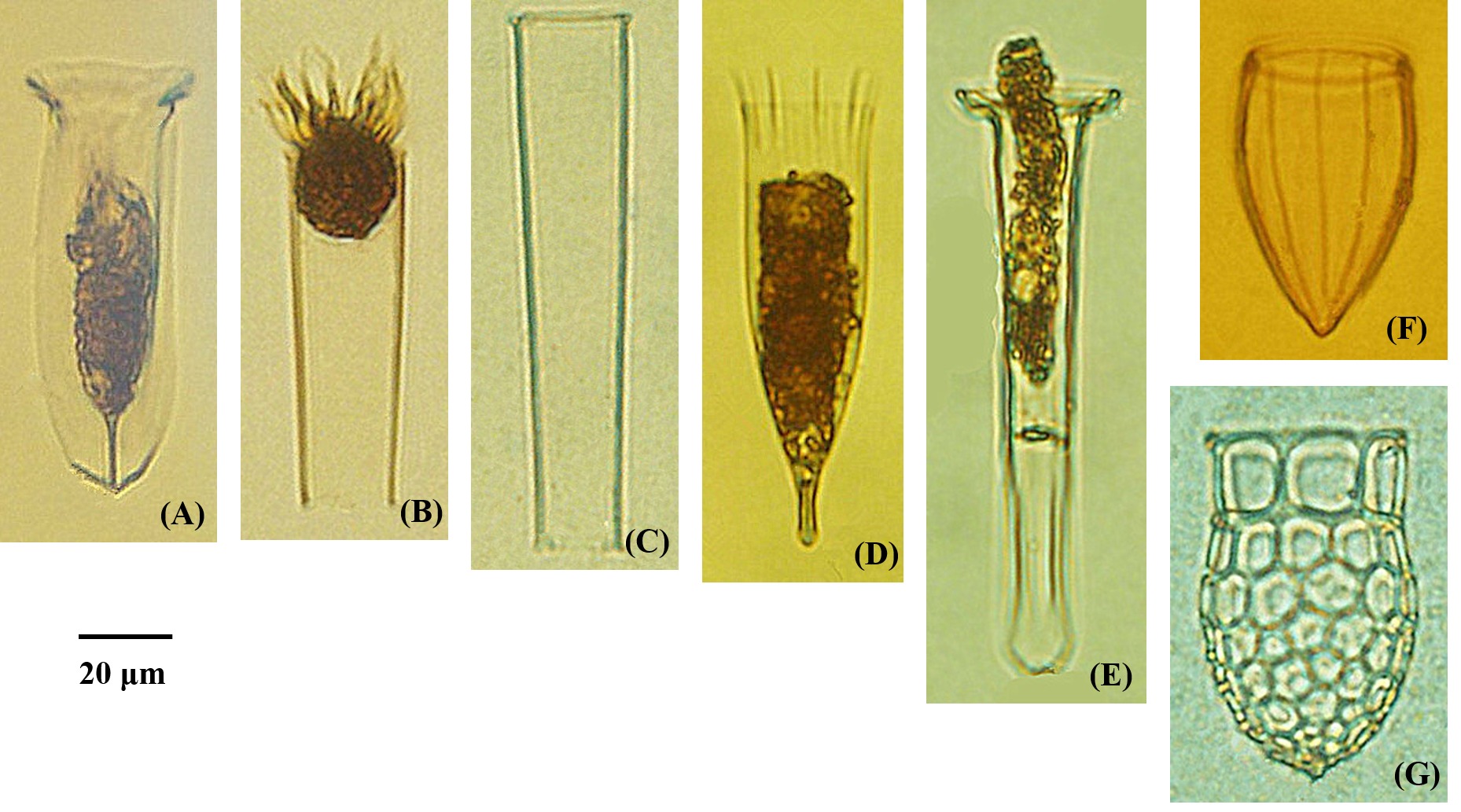

Supplement: Supplementary Figure 3 — Photographs of warm water type I species observed in this study. (A) Amphorides minor; (B) Eutintinnus pacificus; (C) E. tubulosus; (D) Dadayiella ganymedes; (E) Steenstrupiella steenstrupii; (F) Dictyocysta mitra; and (G) Protorhabdonella simplex. [file Image_3.TIF]

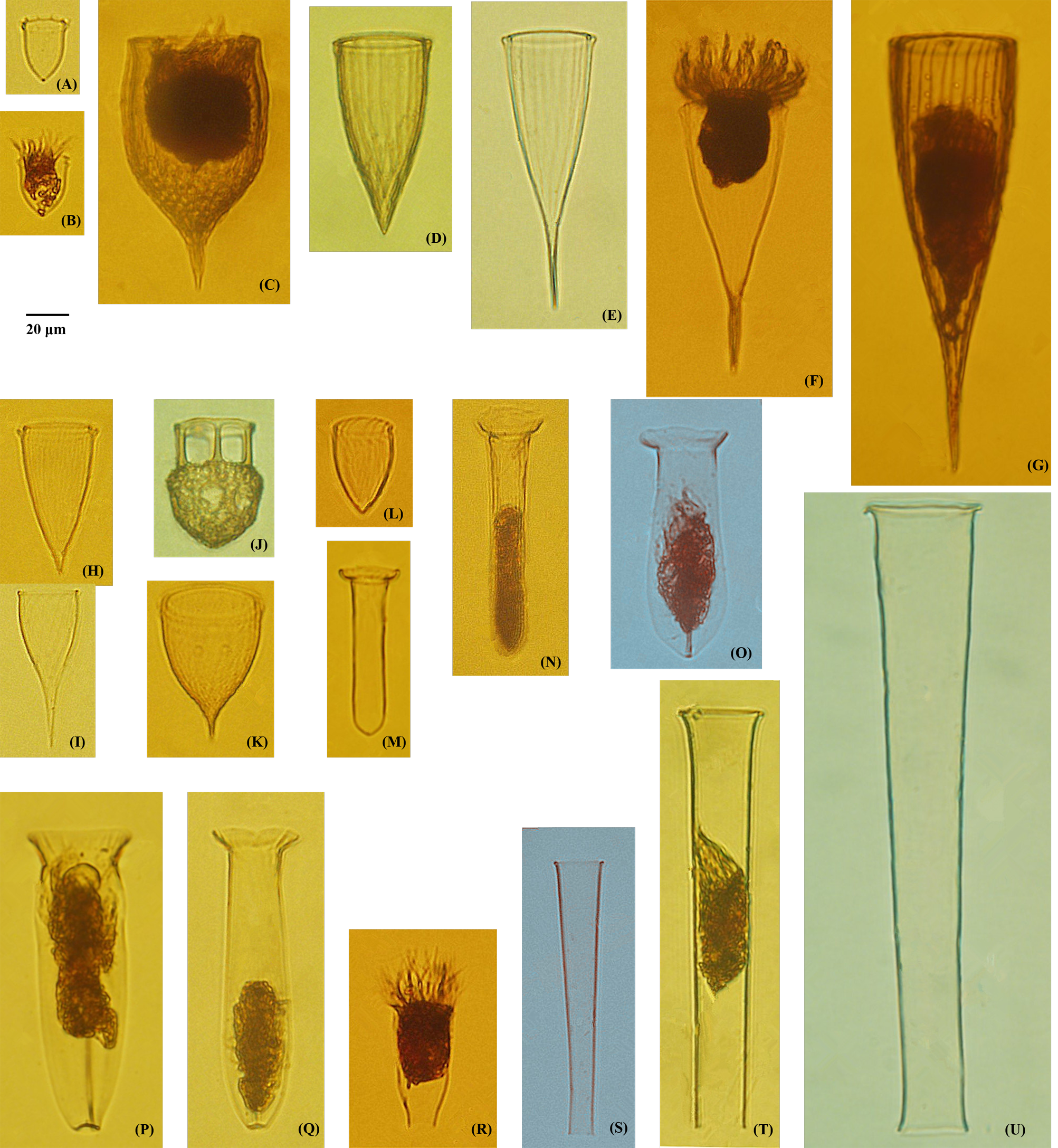

Supplement: Supplementary Figure 4 — Photographs of warm water type II species observed in this study. (A) Acanthostomella minutissima; (B) Ascampbelliella armilla; (C) Epiplocylis constricta; (D) Rhabdonella amor; (E) R. sanyahensis; (F) R. cornucopia; (G) R. elegans; (H) R. indica; (I) R. exilis; (J) Dictyocysta reticulata; (K) Epiplocyloides reticulata; (L) Protorhabdonella curta; (M) Steenstrupiella gracilis; (N) S. robusta; (O) Amphorides amphora; (P) A. quadrilineata; (Q) A brandti; (R) Eutintinnus apertus; (S) E. stramentus; (T) E. lusus-undae; and (U) E. fraknoii. [file Image_4.TIF]

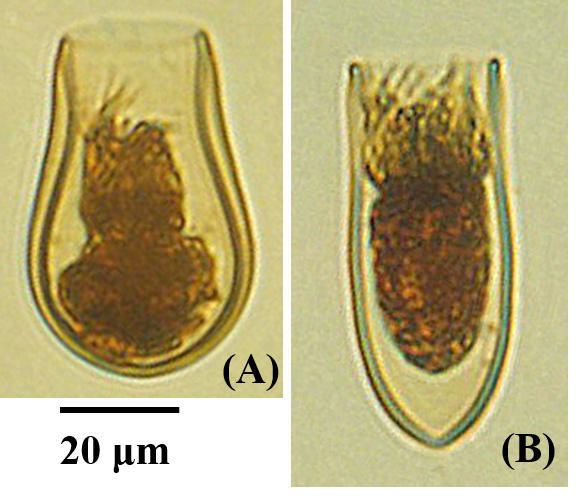

Supplement: Supplementary Figure 5 — Photographs of transition zone species observed in this study. (A) Undella californiensis; (B) U. clevei. [file Image_5.TIF]

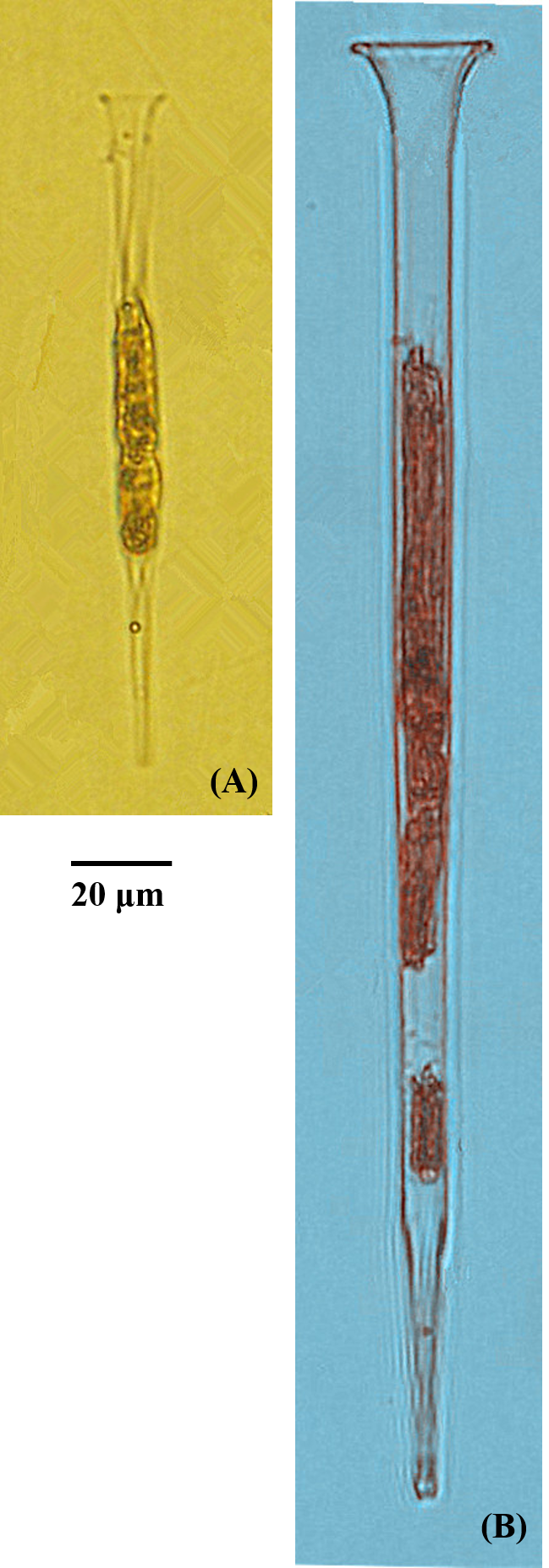

Supplement: Supplementary Figure 6 — Photographs of cosmopolitan species observed in this study. (A) Salpingella faureia; (B) S. cuminata. [file Image_6.TIF]

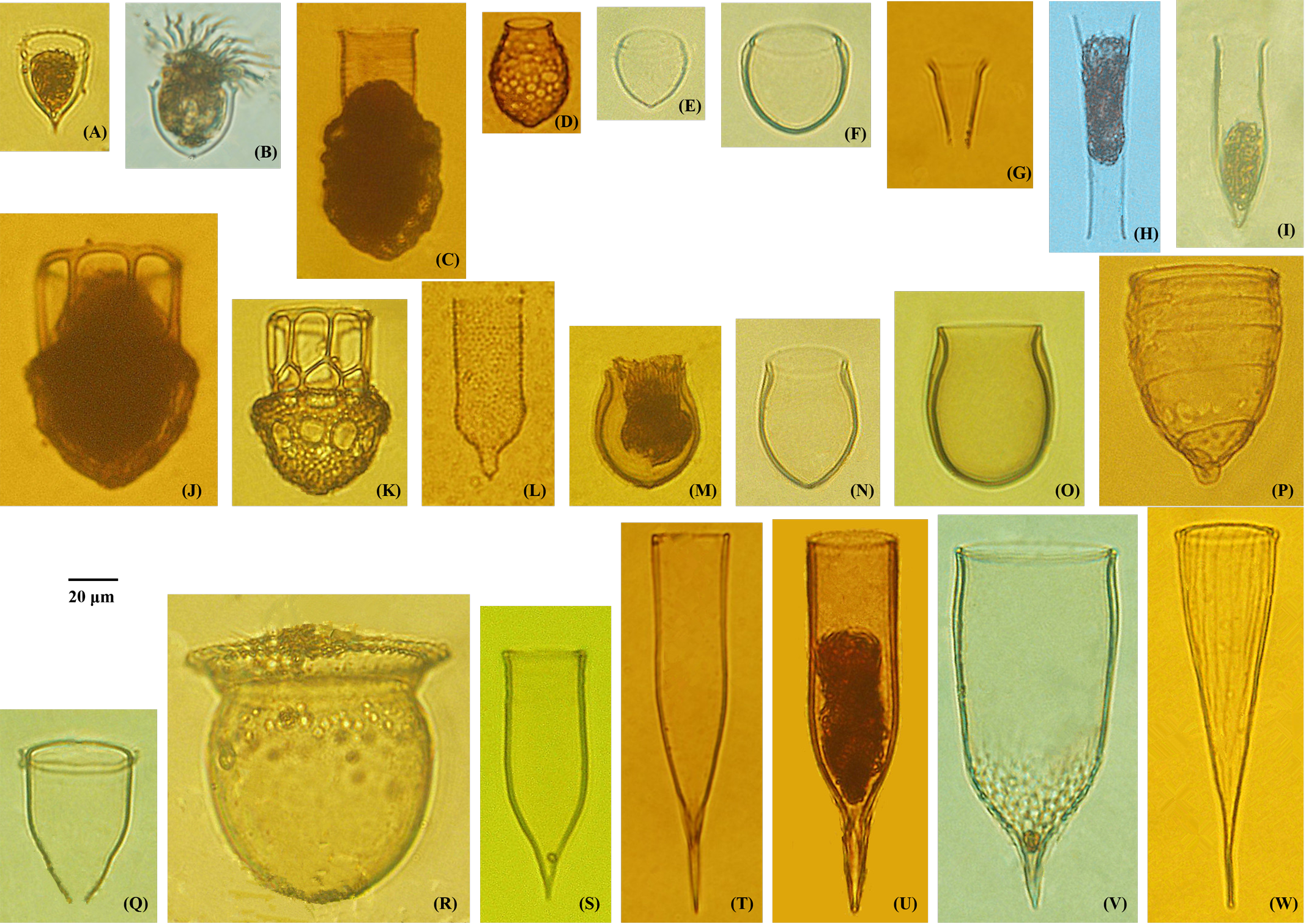

Supplement: Supplementary Figure 7 — Photographs of rare oceanic species observed in this study-part 1. (A) Acanthostomella lata; (B) Ascampbelliella retusa; (C) Codonellopsis morchella; (D) C. contracta; (E) Metacylis sanyahensis; (F) Undella turgida; (G) Eutintinnus haslae; (H) E. macilentus; (I) Amphorellopsis sp.; (J) Dictyocysta polygonata; (K) D. speciosa; (L) Poroecus curtus; (M) Proplectell. perpusilla; (N) P. ostenfeldi; (O) P. claparedei; (P) Coxliella laciniosa; (Q) Petalotricha aperta; (R) P. major; (S) Parundella lachmanni; (T) P. aculeata; (U) P. caudata; (V) Epiplocylis undella; and (W) Protorhabdonella striatura. [file Image_7.TIF]

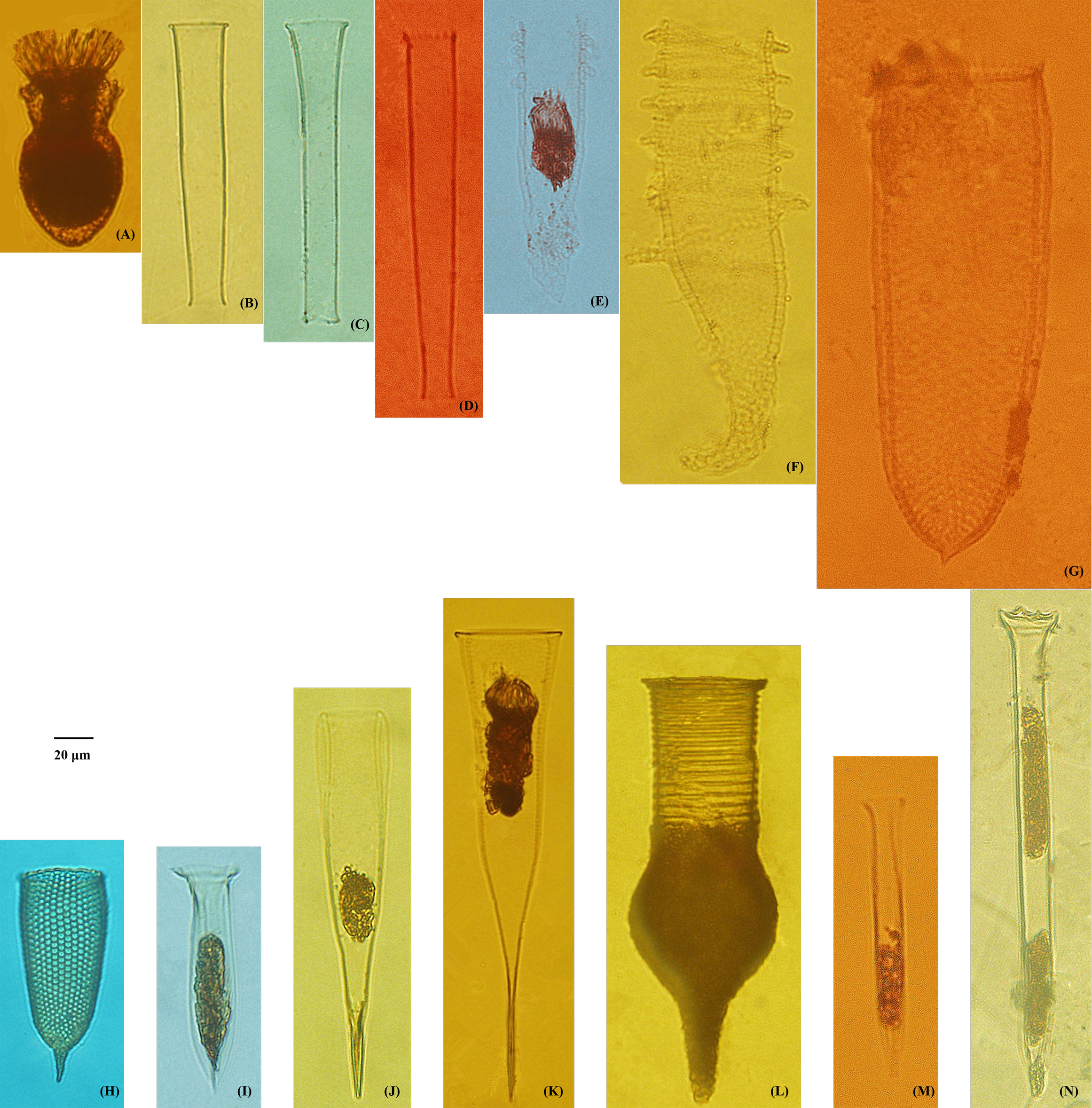

Supplement: Supplementary Figure 8 — Photographs of rare oceanic species observed in this study-part 2. (A) Codonella aspera; (B) Eutintinnus elegans; (C) Eutintinnus sp.; (D) E. turris; (E) C. scalaroides; (F) Climacocylis scalaria; (G) Parafavella denticulata; (H) P. pacifica; (I) Amphorellopsis acuta; (J) Xystonellopsis brandti; (K) Xystonella lanceolata; (L) Codonellopsis meridionalis; (M) Salpingella minutissima; and (N) Salpingacantha unguiculata. [file Image_8.TIF]

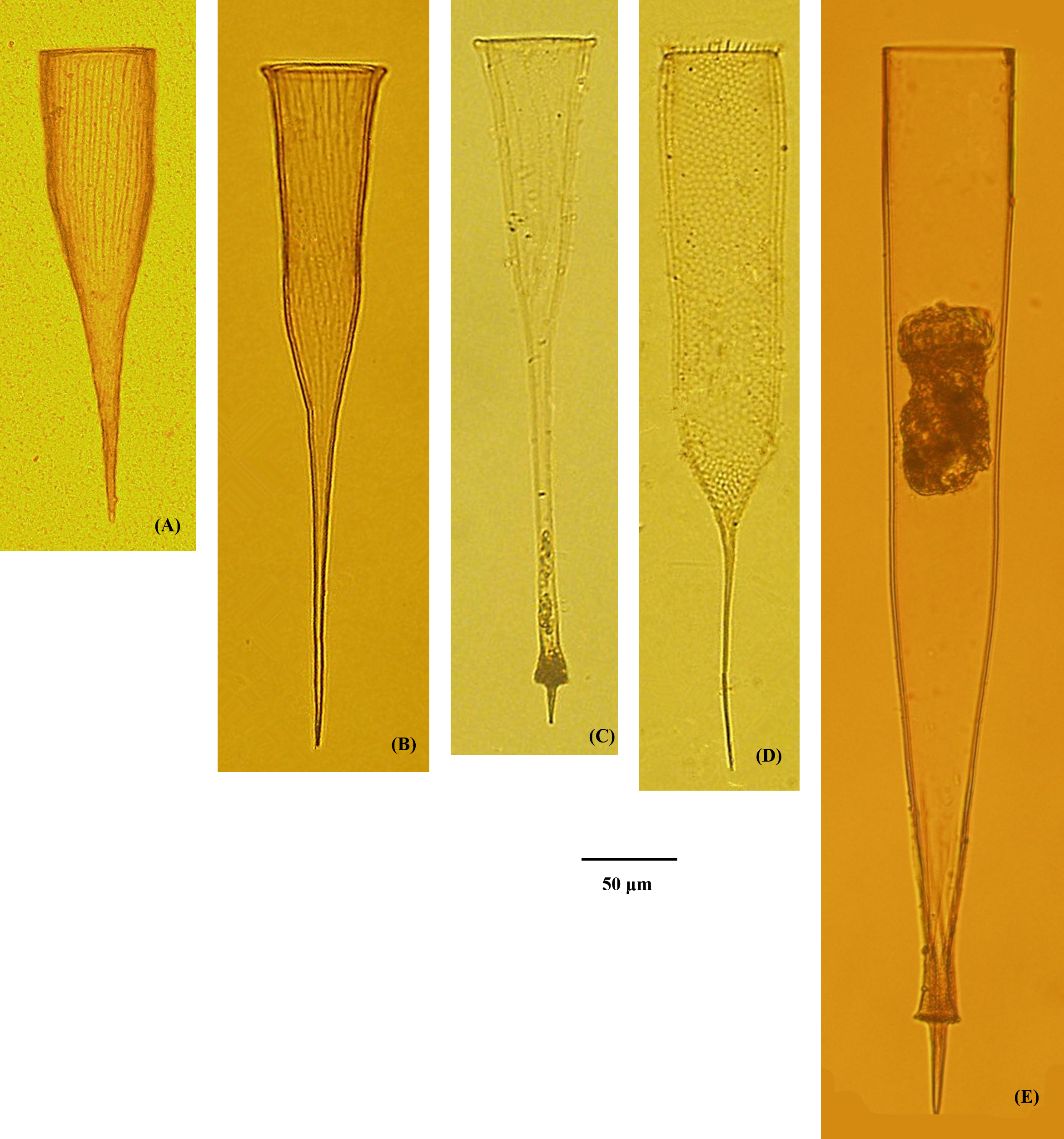

Supplement: Supplementary Figure 9 — Photographs of rare oceanic species observed in this study-part 3. (A) Rhabdonella valdestriata; (B) R. conica; (C) Xystonella treforti; (D) Parafavella promissa; and (E) Xystonellopsis heros. [file Image_9.TIF]

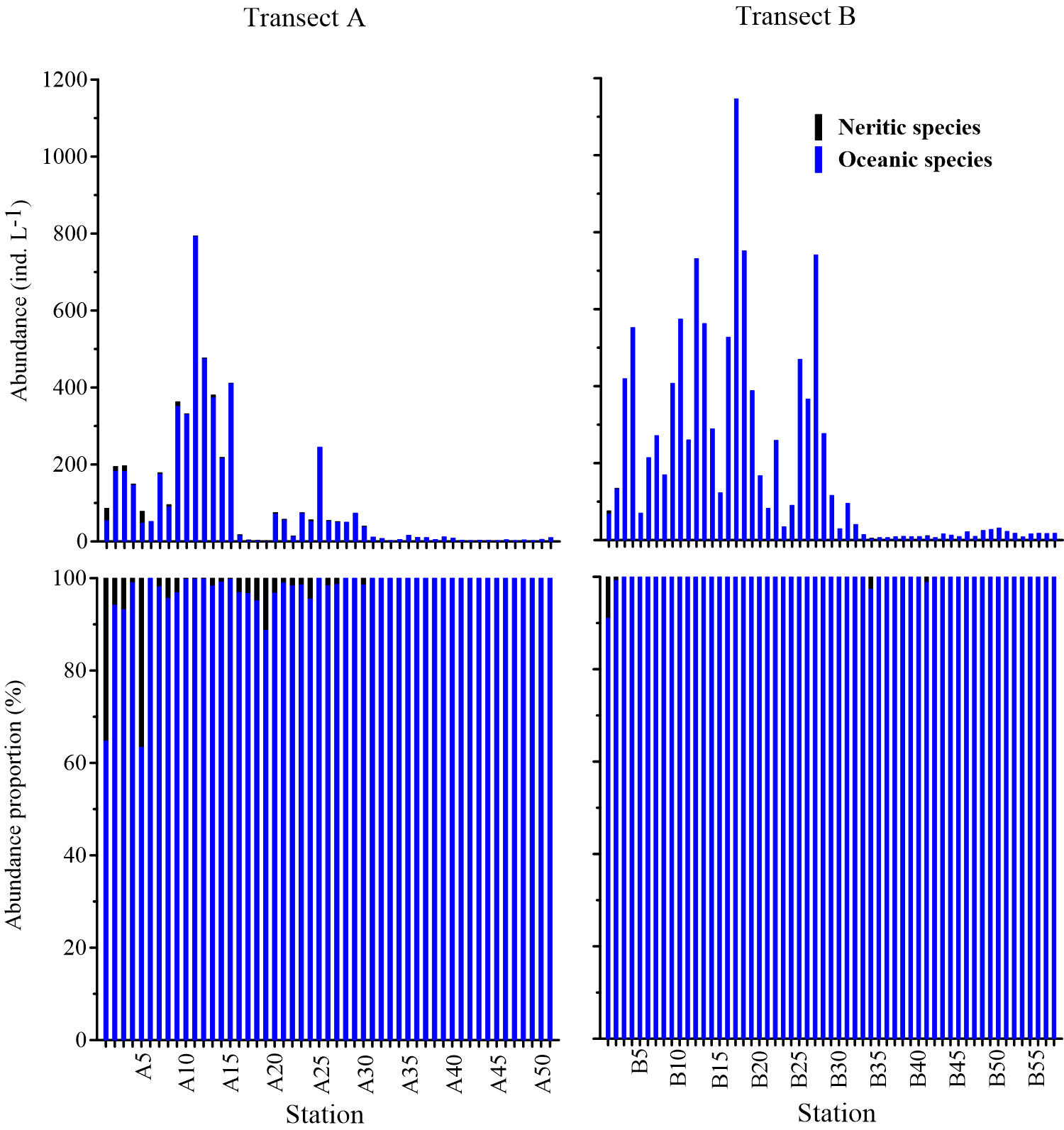

Supplement: Supplementary Figure 10 — Abundance and abundance proportion of neritic and oceanic species at each station. [file Image_10.TIF]

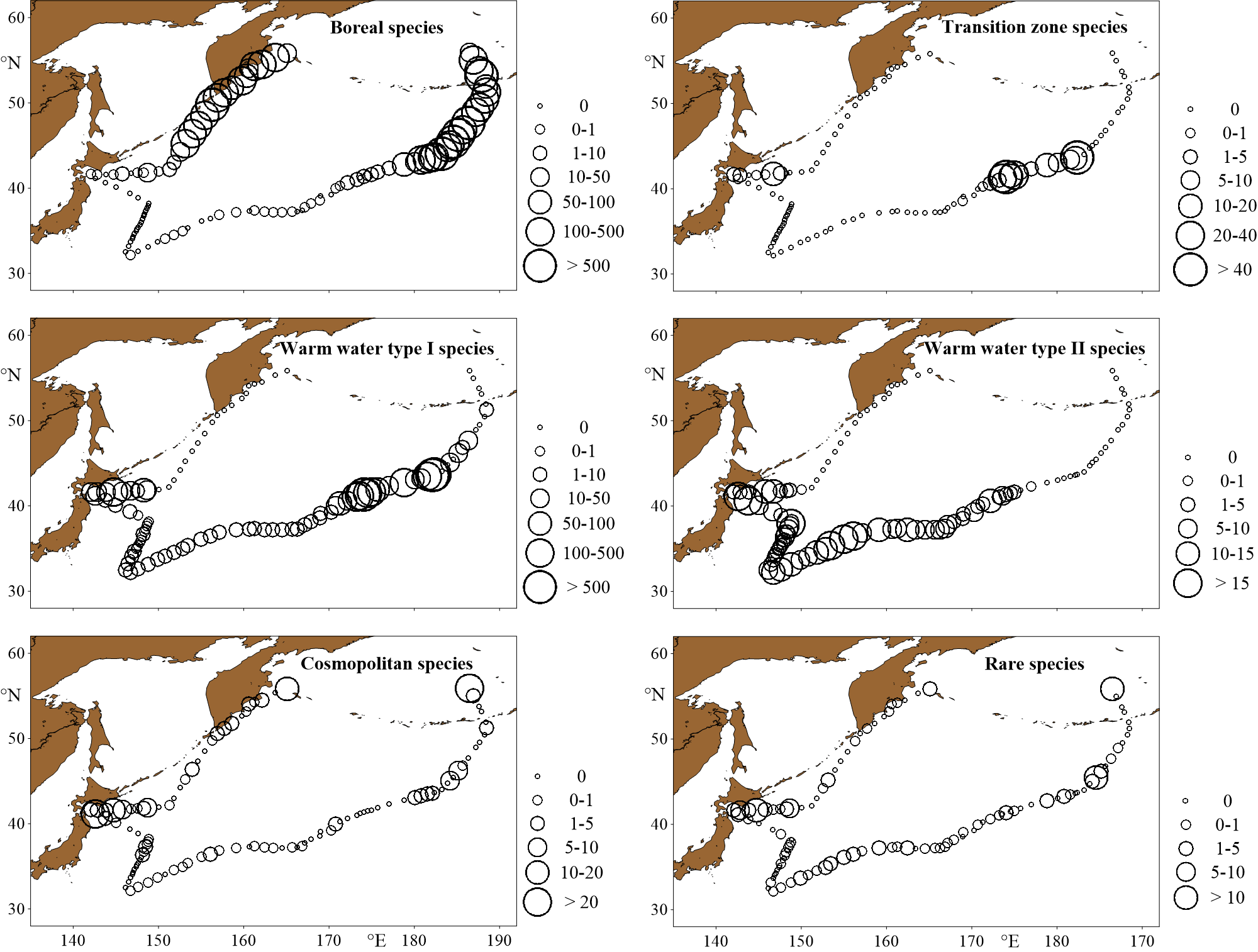

Supplement: Supplementary Figure 11 — Abundance variation of each tintinnid oceanic group in two transects. [file Image_11.TIF]
